# Robotic Ileal Neovaginoplasty for Vaginal Reconstruction: A Report of Two Cases

**DOI:** 10.7759/cureus.113388

**Published:** 2026-07-26

**Authors:** Prakruthi S, Rubina Shanawaz, Mohan Keshavamurthy

**Affiliations:** 1 Urogynaecology and Robotic Surgery, Fortis Hospital, Bengaluru, IND; 2 Urogynaecology, Gynaec-Oncology and Robotic Surgery, Fortis Hospital, Bengaluru, IND; 3 Urology, Uro-Oncology, Andrology, Uro-Gynaecology, Transplant and Robotic Surgery, Fortis Hospital, Bengaluru, IND

**Keywords:** ileal vaginoplasty, minimally invasive gynecology, mullerian anomaly, neovaginoplasty, robotic surgery, vaginal agenesis

## Abstract

Neovaginal reconstruction remains technically challenging in patients with congenital or acquired vaginal agenesis. Ileal segment vaginoplasty is a well-established option because of its favorable mucosal characteristics, natural lubrication, and long-term patency. Robotic-assisted surgery provides enhanced precision, improved ergonomics, and superior access to the deep pelvis, making it well-suited for complex reconstructive procedures. This case series reports two patients with vaginal agenesis who underwent robotic-assisted ileal neovaginoplasty using a standard four-port robotic platform. A 10 cm segment of terminal ileum was isolated with intact mesentery, mobilized, and anastomosed to a surgically created neovaginal canal after pelvic dissection. Both patients had uneventful intraoperative and postoperative courses, with minimal blood loss and operative times of 120 and 165 minutes. Recovery was smooth, with early ambulation and discharge on postoperative day four. At six-month follow-up, both patients had stable neovaginal depth, good cosmesis, and satisfactory functional outcomes without stenosis or significant mucous discharge. Robotic ileal neovaginoplasty appears to be a safe and effective minimally invasive option for vaginal reconstruction in selected patients.

## Introduction

Vaginal agenesis, whether congenital or acquired, represents a challenging problem in reconstructive gynecology. Common indications for neovaginal creation include Mayer-Rokitansky-Küster-Hauser syndrome, disorders of sex development, and selected post-traumatic or post-oncologic defects. Several operative techniques have been described, including McIndoe vaginoplasty, sigmoid vaginoplasty, and intestinal vaginoplasty, each with distinct advantages and limitations [1-6].

Among the available reconstructive options, ileal vaginoplasty remains an attractive technique because it provides a well-vascularized, non-keratinized, and naturally lubricated neovaginal lining with favorable long-term patency. Compared with skin graft-based methods, it avoids donor-site morbidity and may reduce the tendency toward contraction and introital stenosis. In addition, the ileum offers adequate mobility and reliable mesenteric vascularity, allowing the creation of a neovagina with satisfactory anatomical, cosmetic, and functional outcomes. These characteristics make ileal vaginoplasty a valuable option in selected patients requiring durable vaginal reconstruction. Long-term outcome studies of intestinal vaginoplasty have shown acceptable anatomical and functional results in appropriately selected patients [2-4]. More recently, robotic-assisted surgery has expanded the reconstructive armamentarium by improving visualization, dexterity, and suturing capability in the deep pelvis. These technical advantages are particularly relevant in ileal neovaginoplasty, where precise pelvic dissection and secure bowel-to-introitus anastomosis are essential. In this report, two patients with congenital vaginal agenesis underwent robotic ileal neovaginoplasty, and the perioperative technique and early outcomes are described.

## Case presentation

Case 1

A 20-year-old, phenotypic female presented with a desire for vaginal reconstruction and absence of a vaginal canal. Karyotyping revealed a male genotype (46, XY). She had previously undergone bilateral orchidectomy at five years of age after a diagnosis of complete androgen insensitivity syndrome. Following multidisciplinary counseling, robotic ileal neovaginoplasty was planned.

Case 2

A 24-year-old phenotypic female presented with primary amenorrhea and absent vaginal canal. Evaluation was consistent with congenital utero-vaginal agenesis in the Mayer-Rokitansky-Küster-Hauser spectrum in a case of 46, XY. After counseling regarding management options, she opted for robotic ileal neovaginoplasty.

Both patients had no significant comorbidities. Preoperative assessment included a physical examination, pelvic magnetic resonance imaging, hormonal profile, and anesthetic clearance.

Written informed consent was obtained from both patients after discussion of the procedure, risks, benefits, alternatives, and the need for postoperative vaginal mold use and follow-up. Consent for anonymous publication of clinical details and images was obtained.

Surgical technique

Both patients were admitted one day before surgery for bowel preparation and standard preoperative aseptic measures. The procedure, anesthesia plan, expected postoperative course, and postoperative mold care were discussed with the patients and their families.

**Figure 1:**
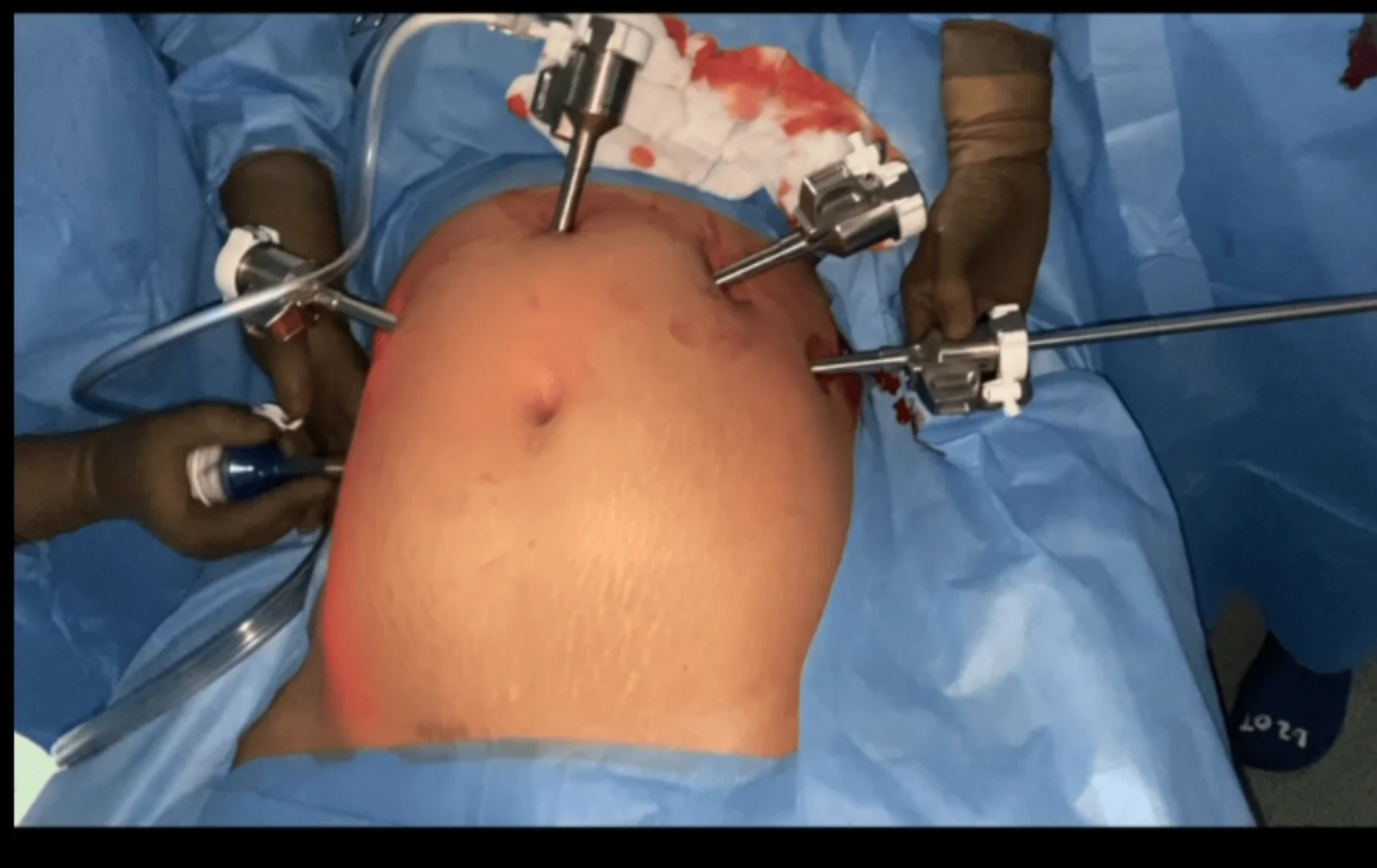
Robotic port placement for robotic ileal neovaginoplasty The ports are placed 5 cm above the umbilicus in a straight line to harvest a suitable segment of ileum.

Under general anesthesia, the patients were placed in lithotomy with steep Trendelenburg. A standard four-port Da Vinci Xi robotic configuration was used, as shown in Figure 1.

**Figure 2:**
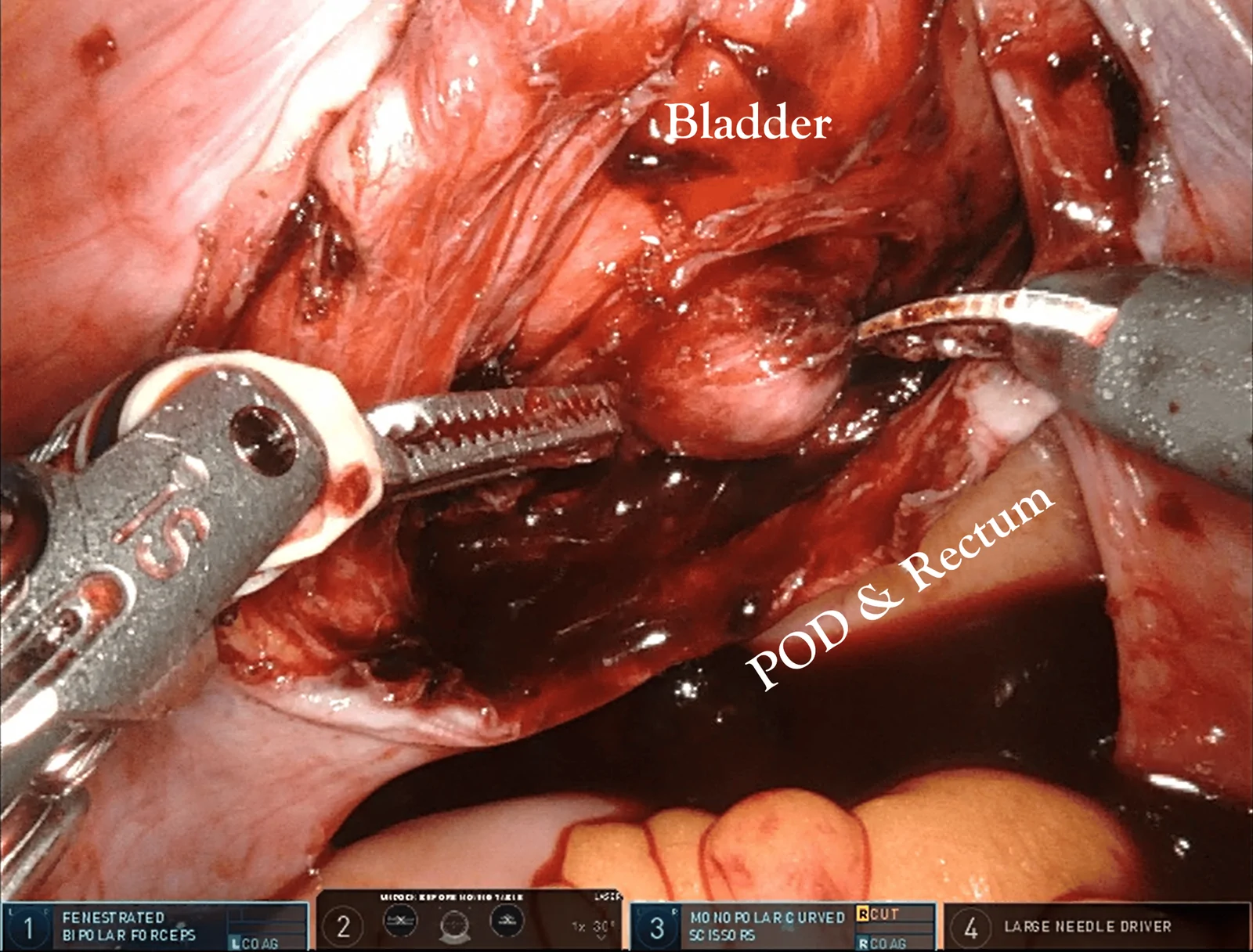
Creation of a neovaginal canal between the bladder and rectum The vaginal dimple is being elevated from the perineum by the vaginal assistant.

A neovaginal canal was created by careful dissection between the bladder and rectum, using a finger as a guide to maintain the correct plane and reduce the risk of rectal injury, as shown in Figure 2.

**Figure 3:**
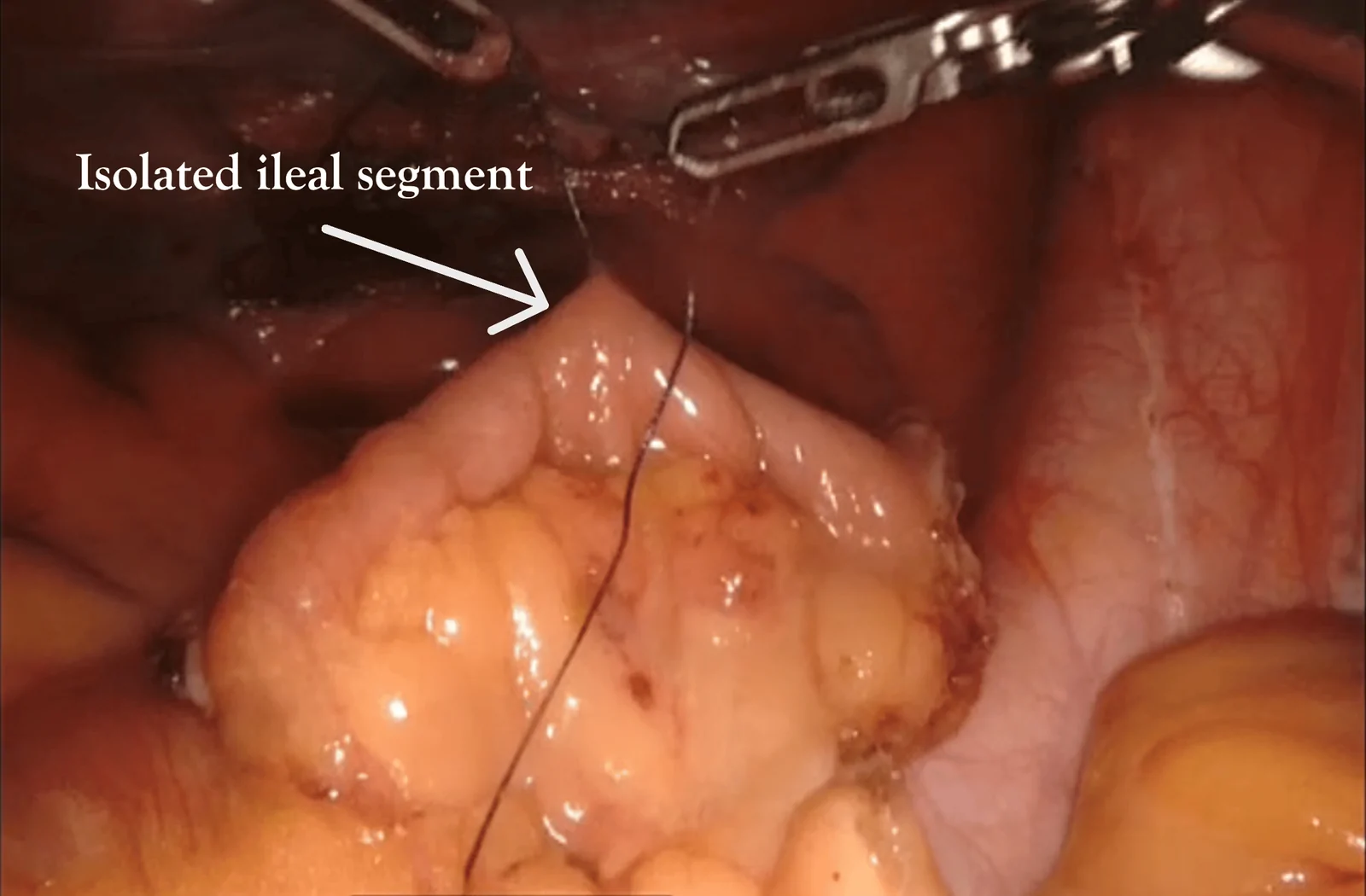
Isolation of the terminal ileal segment with preservation of mesenteric blood supply

A 10 cm segment of terminal ileum, as shown in Figure 3, approximately 15 cm proximal to the ileocecal valve, was identified and mobilized with preservation of its mesenteric blood supply. Bowel continuity was restored using a side-to-side stapled anastomosis. The antimesenteric border of the isolated ileal segment was then incised along its full length. One edge of the opened segment was folded onto itself and sutured with running 2-0 absorbable polyglactin sutures. The segment was subsequently inverted so that the mucosal surface faced inward, while the mesenteric border formed the cranial covering of the neovagina in a “parachute-like” fashion. The fashioned ileal segment was then oriented toward the pelvic floor and brought down to the neovaginal space. Its distal end was anastomosed to the neovaginal canal at the introitus using interrupted 3-0 polydioxanone sutures. A 10 cm segment of terminal ileum, approximately 15 cm proximal to the ileocecal valve, was identified and mobilized with preservation of its mesenteric blood supply. Bowel continuity was restored using a side-to-side stapled anastomosis. The antimesenteric border of the isolated ileal segment was then incised along its full length. One edge of the opened segment was folded onto itself and sutured with running 2-0 absorbable polyglactin sutures. The segment was subsequently inverted so that the mucosal surface faced inward, while the mesenteric border formed the cranial covering of the neovagina in a “parachute-like” fashion. The fashioned ileal segment was then oriented toward the pelvic floor and brought down to the neovaginal space. Its distal end was anastomosed to the neovaginal canal at the introitus using running 3-0 polygluconate V-Loc sutures, which were tightened after complete end-to-end anastomosis. A pelvic drain was placed, and a vaginal mold fashioned from a silicone urinary catheter was inserted to maintain patency and reduce the risk of early stenosis.

Postoperatively, the vaginal mold was maintained in situ for five to seven days. Prophylactic antibiotics were administered, and stool softeners were prescribed to minimize straining. The drain was removed on postoperative day two when output was minimal. Enhanced recovery principles were followed, with oral intake resumed after approximately 18 hours of fasting and early ambulation encouraged.

Results

Both procedures were completed robotically without intraoperative complications. Operative times were 120 and 165 minutes, respectively. Estimated blood loss was less than 100 mL in both cases. Postoperative recovery was uneventful, with oral intake resumed by postoperative day one to two and discharge on postoperative day four.

Vaginal molds were used in the immediate postoperative period to maintain the caliber of the neovagina. At the six-month follow-up, both patients had maintained a vaginal depth of 8.5 to 10 cm, healthy mucosal appearance, and no evidence of stenosis, fistula, or problematic mucous discharge. Both patients reported satisfaction with cosmetic appearance and early functional outcomes.

## Discussion

Ileal neovaginoplasty offers several advantages over skin graft-based and some other reconstructive techniques. The ileal segment provides a vascularized, non-keratinized lining with intrinsic lubrication, which may improve long-term patency and reduce the tendency toward contraction or stenosis. Prior reports of intestinal vaginoplasty have also demonstrated durable anatomical reconstruction and satisfactory functional outcomes in selected patients [2-4].

The robotic platform may further enhance this approach by facilitating dissection in a narrow pelvic space and enabling precise intracorporeal suturing [7]. Improved three-dimensional visualization and instrument articulation are especially useful when creating the neovaginal canal, mobilizing the bowel segment, and performing the ileal anastomosis at the introitus. Previous experience with robot-assisted vaginal construction using the ileum has also demonstrated the technical feasibility of this minimally invasive strategy [8].

The present series adds to the limited literature on robotic ileal neovaginoplasty by demonstrating successful completion of both procedures without intraoperative complications, with minimal blood loss and favorable short-term outcomes. Even so, this technique has important limitations, including higher cost, limited availability, and a substantial learning curve associated with robotic reconstructive surgery. In addition, the small sample size and short duration of follow-up in this series preclude firm conclusions regarding long-term sexual function, durability, and comparative superiority over other established techniques. Larger studies with longer follow-up are needed to better define the role of robotic ileal neovaginoplasty in vaginal reconstruction.

## Conclusions

Robotic ileal neovaginoplasty combines the physiological advantages of an ileal segment neovagina with the technical benefits of robotic surgery. In this report of two cases, the procedure provided satisfactory anatomical reconstruction, favorable early functional outcomes, and minimal perioperative morbidity. It may represent a valuable minimally invasive option for vaginal reconstruction in carefully selected patients and in centers with appropriate expertise.
